# Unusually isolated *Staphylococcus arlettae* in intra-oral sutures - Case series

**DOI:** 10.1099/acmi.0.000555.v4

**Published:** 2023-08-24

**Authors:** Radhika Sunil Kherdekar, Ashutosh Dixit, Ashish Kothari, Kamal Prasad Pandey, Hoshang Advani, Amit Gaurav, Balram Ji Omar

**Affiliations:** ^1^​ Department of Dentistry, Periodontics, All India Institute of Medical Sciences, Rishikesh- 249203, India; ^2^​ Department of Microbiology, All India Institute of Medical Sciences, Rishikesh-249203, India; ^3^​ Department of Biotechnology, Indian Institute of Technology, Roorkee-247667, India

**Keywords:** intra-oral sutures, periodontal, scanning electron microscopy, *Staphylococcus arlettae*, surgical site infection

## Abstract

**Introduction.:**

The human oral cavity comprises various niches such as teeth, gingiva, tongue, soft and hard palate, and various dental prostheses, all inhabited by different bacterial species. Although more than 600 taxa belong to the oral cavity, identifying *Staphylococcus arlettae *, an incompletely understood bacterium, has been rare.

**Methods.:**

Three patients who underwent periodontal flap surgeries were reported with the incidental finding of *S. arlettae* associated with the intra-oral sutures placed. Environmental sampling was performed, to establish the exact source of this bacterium.

**Results.:**

*
Staphylococcus arlettae* was isolated in three patients’ intra-oral sutures. All environmental samples were negative for the presence of the bacterium.

**Conclusion:**

. To this date, no studies have identified such an occurrence of *Staphylococcus arlettae* with intra-oral sutures. Its identification in association with foreign materials, such as sutures, can be considered a potential for surgical site infections and requires further investigation.

## Data Summary

No new data, tools, software or code were generated.

## Introduction

Previously, the coagulase-negative Staphylococci were thought to be non-pathogenic since they lacked the ability to clot blood plasma. They are, however, among the most prevalent organisms to cause bacteremia associated with indwelling devices worldwide. *Staphylococcus arlettae *, first isolated from farm animals' skin and respiratory organs, is a Gram-positive coagulase-negative coccus, non-motile, non-sporulating, occurring singly, in pairs, groups, or short chains [1]. These are facultative anaerobic bacteria and are known to grow exuberantly on Brain Heart Infusion Agar (BHIA) with colonies appearing opaque, yellowish white or beige coloured, with complete margins, 6 to 8 mm in diameter after 2 days of incubation [1]. Although the pathogenicity of this bacterium is not very clearly understood; it has been attributed to the polysaccharide components enabling persistent attachment to foreign materials. Most coagulase-negative organisms are hospital-acquired making them the frontiers of nosocomial infection, adding to morbidity and excess medical costs. Isolation of the *S. arlettae* in the clinical samples has been a rare occurrence, with only a few reports identifying a particular strain in the blood of cardiovascular disease patients [2]. In contrast, others describe their incidence on cell phone surfaces and disused laboratory units [2–4]. Here, we have isolated *S. arlettae* from the intra-oral sutures placed in three patients who underwent treatment with open flap debridement for chronic periodontitis.

## Case report

**Fig. 1.**
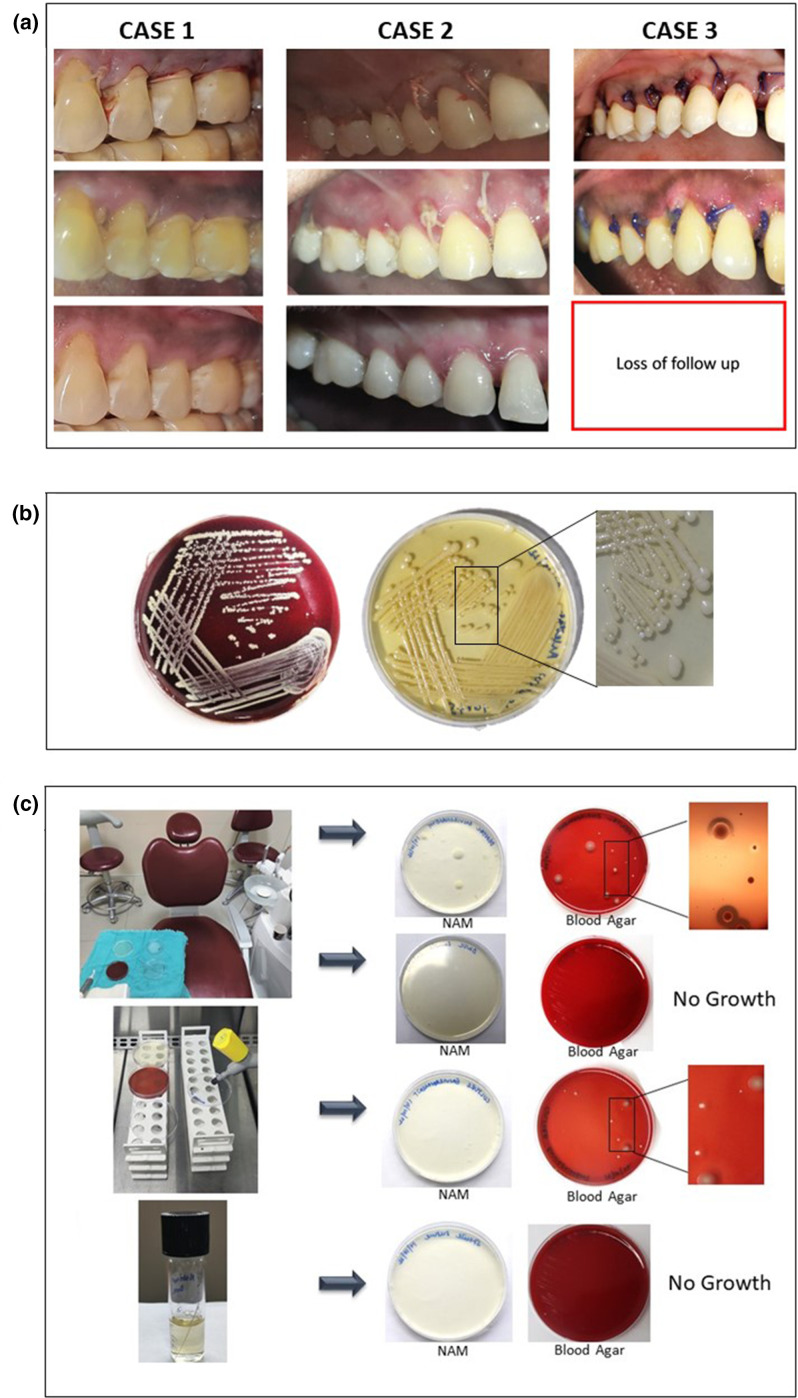
Materials and methods: (a) Clinical presentation of three cases at Day 0 (day of surgery), Day 8 (suture removal) and Day 30 (follow-up) assessing the wound healing. (b) *Staphylococcus arlettae* solation in pure form on blood agar and BHIA (brain heart infusion agar) media, and bacterial morphology by SEM. (c) Environmental sampling on NAM (nutrient agar medium) and blood agar from dental chair – showing β hemolytic organism growth and dental water unit lines – showing no microbial growth, microbiology laboratory workstation (BLST) (biosafety cabinet type-IIA) – showing growth other than *S. arlettae* and sterile suture on incubation – showing no microbial growth).

Three patients, a 61 year-old-male, 41 year-old-female and 24 year old female, presenting to the Department of Dentistry were diagnosed with chronic periodontitis [5]. The persistence of periodontal pockets warranted surgical therapy wherein open flap debridement by raising a Kirkland flap was done for all four quadrants of the oral cavity in a staged approach following the sterile surgical protocol [6]. As the patients were enrolled in an ongoing study for microbiological assessment of the suture sample, the suture on removal was sent for microbiological culture. Those with reported identification of *Staphylococcus arlettae* from their suture samples were included for further study. Since the occurrence of samples positive with *Staphylococcus arlettae* culture was an event by chance, no randomization, blinding or power analysis of sample size was done. All three patients were requested to follow up at 1 month from the time of identification of *Staphylococcus arlettae* in the sample for any signs of fever, pain, or delayed wound healing and to report the incidence of any such untoward experience post-operatively. Wound healing was assessed at the time of suture removal and 1 month post-operatively, using the Early Wound Healing Score [7] (Fig. 1a).

## Bacterial culture of suture samples

The intra-orally placed sutures (MITSU polyglactin 910 sutures, MERIL Life Sciences, Pvt, Ltd) for periodontal flap approximation were aseptically removed on eighth post-surgical day and collected in 5 ml Thioglycolate Broth (HiMedia, Mumbai, India) in sterile tubes. These were vortexed for 2 min. After a serial dilution up to 1 : 10^6^, 0.1 ml aliquot of the final diluted sample was then cultured on Blood Agar (HiMedia, Mumbai, India) and incubated at 37 °C for 24 h. Individual colonies were then sub-cultured and processed immediately as per routine hospital procedure of examination, including bacterial identification, Gram-staining (HiMedia, K001, Mumbai, India), colony morphology characterization by size, shape, texture, opacity and identified by the VITEK two automated identification system, (bioMérieux, Marcy, France). A confirmatory identification using the MALDI-TOF assay (MALDI Biotyper, Bruker) was done for isolated colonies of *Staphylococcus arlettae *. Isolated colonies were also subjected to antibiotic susceptibility assays which were performed using an automated system – VITEK 2 AST cards (bioMérieux, Marcy, France) following the guidelines from Clinical and Laboratory Standards Institute (CLSI), USA [8]. Demographical distribution along with antibiotic susceptibility pattern of three cases included in the present study have been mentioned in the Table 1.

**Table 1.** Summary of data. Demographical distribution along with antibiotic susceptibility pattern of three cases included in the present study

Parameters	Case-1		Case-2		Case-3	
**Age**	61		41		24	
**Gender**	Male		Female		Female	
**Surgical site**	Maxillary		Maxillary		Maxillary	
**Early wound healing score (EHS) on Day 0**	5		5		5	
**Early wound healing score (EHS) on Day 8**	10		8		8	
**Early wound healing score (EHS) on Day 30**	10		10		- (Follow-up pending)	
**Visual Analogue Scale for Pain (VAS score**)	6		3		2	
Antibiotic susceptibility test of the identified *Staphylococcus arlettae* strain^*^	MIC	INT	MIC	INT	MIC	INT
Cefoxitin	NEG	−	POS	+	POS	+
Benzyl penicillin	≥0.5	R	≥0.5	R	≥0.5	R
Oxacillin	1	S	≥4	R	≥4	R
Gentamycin	≤0.5	S	≤0.5	S	≤0.5	S
Ciprofloxacin	≤0.5	S	1	I	1	I
Levofloxacin	0.5	S	2	I	2	I
Erythromycin	≥8	R	≥8	R	≥8	R
Clindamycin	≥4	R	≥4	R	≥4	R
Linezolid	2	S	4	S	4	S
Daptomycin	≤0.12	S	≤0.12	S	≤0.12	S
Teicoplanin	1	S	1	S	2	S
Vancomycin	≤0.5	S	≤0.5	S	≤0.5	S
Tetracycline	≤1	S	2	S	2	S
Tigecycline	≤0.12	S	≤0.12	S	≤0.12	S
Nitrofurantoin	≤16	S	≤16	S	≤16	S
Rifampicin	0.5	S	0.06	S	0.12	S
Trimethoprim/ Sulfamethoxazole	≤10	S	80	S	80	R
**Organism identified in dental plaque sample after 1 week of suture removal**	−		*Staphylococcus gallinarum,* *Streptococcus mitis, Streptococcus oralis*		−	

*VITEK two automated identification system, (bioMérieux, Marcy, France).

I, Intermediate resistant; INT, Interpretation; MIC, Minimum inhibitory concentration; R, Resistant; S, Sensitive.

## Environmental sampling

In order to eliminate the possibility of bacterial contamination from the environment at any of the several steps in sample processing, samples representing the environment were taken for isolation of *S. arlettae *. Samples representing the dental chair environment and inoculation environment in the microbiology laboratory were taken by the settle plate method [9]. Water from dental water unit was inoculated in blood and nutrient agar plates. These plates were then incubated at 37 °C for 24 h and observed for growth. The thioglycolate broth bottles and sterile saline bottles for dilution were compared for optical density before and after incubation at 37 °C for 24 h to check for any contaminant within [10]. Sterile sutures from the same batch of manufacturing as those used for the surgeries were inoculated in 5 ml of nutrient broth and observed for growth after incubation at 37 °C for 24 h (Fig. 1b, c).

## Bacterial culture of patient plaque sample

Supra-gingival plaque was collected from the patients with reported incidental findings of *S. arlettae* at their subsequent visit to the OPD (about 1 week) using sterile curettes and placed in a sterile bottle with 5 ml thioglycolate broth. The bottle was vortexed for 2 min, followed by inoculation of 0.1 ml of the solution on blood agar and nutrient agar plates by intermittent heating and streak method. After incubation, the culture plates were observed for growth. Based on colony morphology, colonies similar to that of *S. arlettae* were further sub-cultured and subjected to identification by the VITEK two automated identification system, (bioMérieux, Marcy, France).

## SEM of sutures – *in vitro* biofilm production

**Fig. 2.**
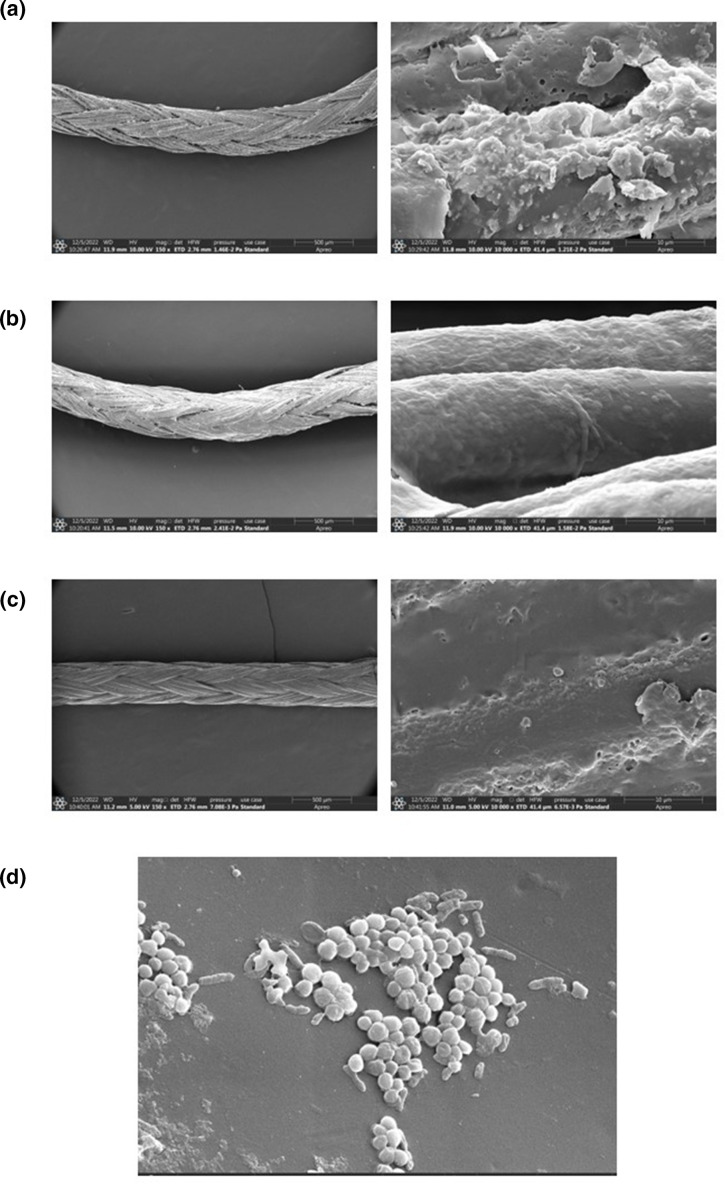
Scanning Electron Microscopy (SEM) images of suture samples with *S. arlettae* biofilm formation: (a) Resorbable chlorhexidine coated polyglycolic-lactic acid (PGLA) suture under 150× and 10000× magnification showing biofilm formation. (b) Resorbable non coated PGLA suture under 150× and 10000× magnification showing biofilm formation. (c) Control (sterile suture) under 150× and 10000× magnification. (d) SEM image of *S. arlettae* colonies at 10000× magnification.

An *in vitro* biofilm production was carried out by placing 1-inch-long sterile suture samples in pure culture of *S. arlettae* and then subjected to scanning electron microscopy (SEM) to get detailed morphological evidence at submicron level. Briefly, isolated sutures were collected and immediately fixed with a phosphate buffer saline solution containing 2 % (vol/vol) formaldehyde for 24 h. Fixed sutures were gradually dehydrated using a series of ethanol gradients (10, 20, 30, 40, 50, 60, 70, 80, 90, and 100%). Sutures were fixed for 20 min at each ethanol gradient. Next, sutures were sputter coated with a layer of gold and observed under scanning electron microscope (Aprio, Thermo Fisher Scientific, USA) (Fig. 2).

## Results

Samples of the three patients yielded an incidental identification of *S. arlettae* from cultured suture samples at different time intervals. All of the environmental samples collected were negative on culture for *S. arlettae *. Furthermore, we performed antibiotic susceptibility testing, and the results are shown in Table 1. Dental plaque samples collected from the same site as the prior suture failed to show the presence of *S. arlettae* after approximately 1 week of suture removal. Other organisms identified included *Staphylococcus gallinarum *, which is very similar to *S. arlettae *, except that they are positive for cellobiose and urease reactions [1]. One of the patients reported gnawing pain and discomfort up to 4 days post-operatively. On examination and evaluation of the healing of the surgical site, no apparent inflammation or deviation from standard healing patterns was present. On 1 month follow-up, these patients showed satisfactory healing, with no reported fever, wound infection or pain during the healing period. Oral hygiene status was good for all three patients. Finally, our SEM results showed high biofilm formation with both types of sutures as compared to the control.

## Discussion

It is apparent that *S. arlettae* has a predilection for foreign material surfaces, even though the specific virulence factors of the bacterium are not as well established as they are in *Staphylococcus aureus* [11]. The literature for its occurrence in human samples is sparse, from being considered as normal skin commensals to being associated with different types of infections especially where a large number of antibiotics have been used [2, 3, 12–14]. This makes its role in surgical site infections questionable. It has been considered as an emerging opportunistic pathogen with an antibiotic resistance profile similar to that of the prominently pathogenic Staphylococcus species – *S. aureus* and *Staphylococcus epidermidis* and virulence factors similar to *Staphylococcus hemolyticus* and *Staphylococcus saprophyticus* [4]. In the past few decades, coagulase-negative Staphylococci have been recognized as one of the important nosocomial pathogens and they also ranked among the top two most frequently isolated organisms in hospital wards and ICUs from the central line associated-bloodstream infections [15].

To the best of our knowledge, this is the first incidence of *S. arlettae* to be isolated from intra-oral samples associated with sutures. Previously, in a multi-centric study which determined the contamination of water in dental unit reservoirs from 107 dental unit reservoir samples located in dental surgeries of public health centres, *S. arlettae* was reported with an incidence rate of 1 (0.93 %) [16]. However, in our setting, we failed to identify any bacterial growth associated with the water samples taken from the dental water unit lines. Clinically, none of the patients could be identified with any significant morbidity attributable to the presence of this bacterium. Whether this bacterium is associated with disease or worsening of periodontal state is still a missing piece of the puzzle and requires further studies.

However, by understanding the microbiological characteristics of *S. arlettae *, we can opinionate on its occurrence in the oral cavity in association with intra-oral sutures as follows:

Virulence factors of this species include specific bacterial binding to extracellular matrix molecules such as laminin, fibronectin, vitronectin, fibrinogen, collagen and others by means of various cell wall proteins [17]. Microscopically identified fimbriae-like structure on this bacterium by electron-microscopy may also be considered as an important role in attachment to foreign materials in the host [18]. Isolating *S. arlettae* from intra-oral sutures and its absence in subsequent plaque samples or environment points to an important clue that it is probably present as a commensal in detectably low numbers, kept in check by the host’s immunity under normal conditions. In the presence of any foreign implantable material, it may exhibit exuberant growth and form a biofilm. The tendency of *S. arlettae* to be associated with foreign materials may result in an increased probability of surgical site infections, causing morbidity to the patient. In our study, one of the three patients reported of persistent gnawing pain at the surgical site and discomfort post-operatively. Pain is however a subjective parameter with varying degrees of assessment and its causative factors can be many, such as surgical manipulation, post-operative care, and therefore difficult to be identified. Also, healing was assessed only clinically, which overlooks the possibility of altered healing at histologic levels.

In our study, the suture material was removed at eighth day post-operatively. However, the resorbable nature of the suture might be convincing to certain clinicians and patients alike to let the suture stay to resorb on its own for longer periods of time. This, however may be disastrous in a situation where such non-coagulase-positive Staphylococci are adherent onto the suture material, allowing its port of entry into the adjacent tissues, especially in an added disadvantage of immune-compromised state of the host. This could be worsened by the presence of multi-drug resistant strains of this species. However, further studies are required to prove the pathogenicity of this bacterium in regard to the oral environment.

Another important point to be stressed is the use of chemical plaque control post-operatively in patients undergoing periodontal flap surgery. The compromised mechanical plaque control that follows the periodontal surgery can increase the plaque formation on the suture materials. An antimicrobial oral rinse such as 0.2 % chlorhexidine digluconate remarkably reduces the bacterial load on the suture materials [19]. Studies have shown that chlorhexidine is effective in reducing coagulase-negative bacterial counts by up to 63.2 % and, thus, should be routinely prescribed to keep pathogenic bacterial counts under control [20]. Chlorhexidine coated sutures with increased drug release locally can add to the benefit of the chemical plaque control and is evident from the SEM results of our study as lesser biofilm formation occurred with the use of coated sutures.

## Conclusion

Coagulase-negative Staphylococci are emerging as opportunistic pathogens and their occurrence on intra-orally placed sutures is an alarming sign, considering the possibility of bacteremia. Duration of sutures *in situ* for wound approximation should be as brief as possible and plaque control in the post-surgical healing phase should be emphasized on. We also urge readers to avoid dismissing the atypically cultured bacteria as commensals or contaminants too easily and encourage discussion to improve our understanding of the microbiological world.

## References

[1] Schleifer KH, Kilpper-Bälz R, Devriese LA (1984). *Staphylococcus arlettae* sp. nov., *S. equorum* sp. nov. and *S. k1oosii* sp. nov.: three new coagulase-negative, novobiocin-resistant species from animals. Syst Appl Microbiol.

[2] Dinakaran V, Shankar M, Jayashree S, Rathinavel A, Gunasekaran P (2012). Genome sequence of *Staphylococcus arlettae* strain CVD059, isolated from the blood of a cardiovascular disease patient. J Bacteriol.

[3] Kurli R, Chaudhari D, Pansare AN, Khairnar M, Shouche YS (2018). Cultivable microbial diversity associated with cellular phones. Front Microbiol.

[4] Lavecchia A, Chiara M, Manzari C, Trotta M, Marzano M (2018). Draft genome sequences of three novel *Staphylococcus arlettae* strains isolated from a disused biological safety cabinet. Microbiol Resour Announc.

[5] Tonetti MS, Greenwell H, Kornman KS (2018). Staging and grading of periodontitis: framework and proposal of a new classification and case definition. J Periodontol.

[6] Newman MG, Takei H, Klokkevold PR, Carranza FA (2018). Newman and Carranza’s Clinical Periodontology.

[7] Marini L, Rojas MA, Sahrmann P, Aghazada R, Pilloni A (2018). Early wound healing score: a system to evaluate the early healing of periodontal soft tissue wounds. J Periodontal Implant Sci.

[8] CLSI C (2016). Performance standards for antimicrobial susceptibility testing. Clin Lab Stand Inst.

[9] Pasquarella C, Pitzurra O, Savino A (2000). The index of microbial air contamination. J Hosp Infect.

[10] Koch AL (1970). Turbidity measurements of bacterial cultures in some available commercial instruments. Anal Biochem.

[11] Huebner J, Goldmann DA (1999). Coagulase-negative staphylococci: role as pathogens. Annu Rev Med.

[12] Andreis SN, Perreten V, Schwendener S (2017). Novel Β-Lactamase BLA ARL in *Staphylococcus arlettae*. MSphere.

[13] Teeraputon S, Santanirand P, Wongchai T, Songjang W, Lapsomthob N (2017). Prevalence of methicillin resistance and macrolide-lincosamide-streptogramin B resistance in *Staphylococcus haemolyticus* among clinical strains at a tertiary-care hospital in Thailand. New Microbes New Infect.

[14] Medeiros DNM, Mafra ACCN, Souza DC de, Troster EJ (2022). Epidemiology and treatment of sepsis at a public pediatric emergency department. Einstein.

[15] Weiner-Lastinger LM, Abner S, Edwards JR, Kallen AJ, Karlsson M (2020). Antimicrobial-resistant pathogens associated with adult healthcare-associated infections: summary of data reported to the National Healthcare Safety Network, 2015–2017. Infect Control Hosp Epidemiol.

[16] Szymańska J, Sitkowska J (2013). Bacterial contamination of dental unit waterlines. Environ Monit Assess.

[17] Crossley KB, Jefferson KK, Archer GL, Fowler VG (2009). Staphylococci in human disease.

[18] Veenstra GJ, Cremers FF, van Dijk H, Fleer A (1996). Ultrastructural organization and regulation of a biomaterial adhesin of *Staphylococcus* epidermidis. J Bacteriol.

[19] Heitz F, Heitz-Mayfield LJA, Lang NP (2004). Effects of post-surgical cleansing protocols on early plaque control in periodontal and/or periimplant wound healing. J Clin Periodontol.

[20] Koscova J, Hurnikova Z, Pistl J (2018). Degree of bacterial contamination of mobile phone and computer keyboard surfaces and efficacy of disinfection with chlorhexidine digluconate and triclosan to its reduction. Int J Environ Res Public Health.

